# Soluble insulin analogs combining rapid- and long-acting hypoglycemic properties – From an efficient *E*. *coli* expression system to a pharmaceutical formulation

**DOI:** 10.1371/journal.pone.0172600

**Published:** 2017-03-15

**Authors:** Diana Mikiewicz, Anna Bierczyńska-Krzysik, Agnieszka Sobolewska, Dorota Stadnik, Monika Bogiel, Monika Pawłowska, Anna Wójtowicz-Krawiec, Piotr A. Baran, Natalia Łukasiewicz, Agnieszka Romanik-Chruścielewska, Iwona Sokołowska, Jacek Stadnik, Piotr Borowicz, Grażyna Płucienniczak, Andrzej Płucienniczak

**Affiliations:** Institute of Biotechnology and Antibiotics, Starościńska 5, 02–516 Warsaw, Poland; Stellenbosch University, SOUTH AFRICA

## Abstract

The discovery of insulin led to a revolution in diabetes management. Since then, many improvements have been introduced to insulin preparations. The availability of molecular genetic techniques has enabled the creation of insulin analogs by changing the structure of the native protein in order to improve the therapeutic properties. A new expression vector pIBAINS for production of four recombinant human insulin (INS) analogs (GKR, GEKR, AKR, SR) was constructed and overexpressed in the new *E*. *coli* 20 strain as a fusion protein with modified human superoxide dismutase (SOD). The SOD gene was used as a signal peptide to enhance the expression of insulin. SOD::INS was manufactured in the form of insoluble inclusion bodies. After cleavage of the fusion protein with trypsin, the released insulin analogs were refolded and purified by reverse-phase high performance liquid chromatography (RP-HPLC). Elongation of chain A, described here for the first time, considerably improved the stability of the selected analogs. Their identity was confirmed with mass spectrometric techniques. The biological activity of the insulin derivatives was tested on rats with experimental diabetes. The obtained results proved that the new analogs described in this paper have the potential to generate prolonged hypoglycemic activity and may allow for even less frequent subcutaneous administration than once-a-day. When applied, all the analogs demonstrate a rapid onset of action. Such a combination renders the proposed biosynthetic insulin unique among already known related formulations.

## Introduction

**Fig 1:**
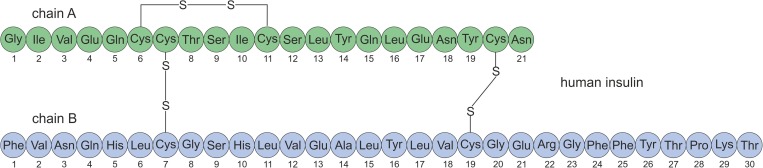
The schematic structure of human insulin.

Human insulin is a hormone produced in the β-cells of the pancreatic islets [1], responsible for glucose metabolism regulation. The amino acid structure of porcine insulin was determined in the early 1950s by Frederick Sanger [2–4]. Subsequently, the elucidation of the chemical structure of human insulin by Nicol and Smith [5] followed. The first biosynthetic human insulin was genetically engineered and produced in *E*. *coli* in 1970s by Arthur Riggs and Keiichi Itakura at the Beckman Research Institute of the City of Hope in collaboration with Herbert Boyer at Genentech [6,7]. Human insulin is a polypeptide with a molecular mass of 5808 Da. The hormone consists of two chains: chain A (21 amino acids) and chain B (30 amino acids) connected by two disulphide bridges. Additionally, chain A contains an intrachain disulfide bond (Fig 1) [8].

An insulin deficit in an organism or a lack of cell response to the produced hormone leads to diabetes mellitus. This usually incurable illness manifests as high blood sugar (hyperglycemia). Chronic hyperglycemia may lead to disturbance in action and the incapacity of various organs including kidneys, heart, blood vessels, the nervous system and retina [9]. Recombinant human insulin introduced as a basic treatment for patients suffering from diabetes in the 1980s did not, however, mimic the physiological profile of endogenous hormone release [10]. The protein, available for clinical use, has been produced by recombinant DNA technology in *E*. *coli* or yeast as a fusion protein, and then converted to the functional hormone by selected enzymes [11,12]. The drug exhibits slower action over a longer period of time than insulin secreted by the pancreas in response to increased blood glucose levels. For this reason, several insulin analogs have been developed to improve glycemic control in diabetic patients. Changes in the insulin structure through chemical or molecular biological modifications were shown to lead to variations in its pharmacokinetics and bioavailability [13].

The drug is now available in the form of rapid-acting, short-acting, intermediate-acting, long-acting Neutral Protamine Hagedorn (NPH) formulations [14], premixed formulations of NPH and regular human insulin. Rapid-acting insulin analogs enable larger amounts of active monomeric insulin to be available for postprandial (after meal) injections. Their onset of action is from 5 to 15 min after subcutaneous injection, they have a faster and greater peak action and are active for 4 to 6 h [15,16]. Here, the changes introduced into the insulin structure involve solely one or two amino acid alterations to weaken the drug’s tendency to self-association and facilitate its absorption [17]. Currently, insulin aspart (Novo Nordisk), insulin lispro (Eli Lilly) and insulin glulisine (Sanofi-Aventis) are commercially available. Short-acting formulations include regular human insulin such as Humulin (Eli Lilly) or Novolin (Novo Nordisk) which begin working within 30 min and are active for about 5 to 8 h. The exogenous hormone in pharmaceutical formulations is a hexamer. Its further dissociation into dimers and subsequently to monomers is required before filtering into the bloodstream and evoking therapeutic action. To maintain a basic level of insulin and to avoid multiple injections, a number of attempts were made to prolong the duration of action of insulin. Hence, intermediate-acting NPH insulin was formulated. Its onset ranges from 1 to 2 h and duration from 18 to 24 h [18]. The addition of protamine, in a molar ratio 1:6, to a regular insulin resulted in a slower release from subcutaneous tissue and an extended time of action [19]. Another common principle used to prolong the action of the hormone is to shift the isoelectric point of human insulin from pH 5.4 towards a more neutral pH by addition of positively charged amino acids to polypeptide chain B. As a result, the insulin remains less soluble at the neutral pH of the injection site, forms microprecipitates at the physiological, neutral pH and then is gradually, slowly released into the blood, giving a long-term therapeutic level [20]. One long-acting analog is insulin glargine (Lantus, Sanofi-Aventis). It is indicated for subcutaneous administration once daily at bedtime in adult and pediatric patients. The structure of insulin glargine differs from regular human insulin by the substitution of glycine for asparagine at position A21 and by the addition of two arginine residues to the C-terminus of chain B. These modifications lead to slower, prolonged drug absorption than regular insulin [21,22]. Another drug, available for commercial use, is insulin detemir (Levemir, Novo Nordisk) with a C14 fatty acid side chain added at position B29. The modification enables detemir to bind with albumin reversibly and to form multimeric complexes within subcutaneous tissue, which prolongs its duration of action [23,24]. The latest ultralong-acting insulin analog to be marketed is insulin degludec (Tresiba, Novo Nordisk). The structure of this drug is characterized by the deletion of the B30 threonine and the addition of a 16-carbon fatty diacid to B29 lysine with a glutamic acid as a spacer. This structure promotes formation of multihexamers in subcutaneous tissue, resulting in a long, flat, extended insulin profile [25,26]. Long-acting analogs cover insulin needs for about 24 h. Premixed insulins and insulin co-formulations are a combination of a rapid-acting insulin analog and its intermediate-acting protamine suspension or a distinct, basal analog, respectively. To name a few, biphasic insulin aspart [27], degludec/insulin aspart [28] or detemir/insulin aspart [29] can be mentioned. The drugs assure basal insulin supplementation and achievement of prandial glycemic coverage. Various proportions of the mixtures are available [19]. More detailed reviews on insulin formulations can be found elsewhere [12,14,30,31].

Despite the improvement in insulin therapy, the available formulations do not ensure ideal, parallel to B-cells, insulin secretion, thus triggering further research on molecules with minimal modification and desirable kinetics. So far, the proposed insulin medicaments are mainly based on insulins altered in chain B. The influence of chain A composition on insulin aggregation properties is less recognized. The aim of our research was to create new insulin analogs with long-acting properties. The analogs were designed to possess additional lysine and/or arginine at the C-terminus of chain B and small, neutral amino acids including alanine, glycine, serine at the C-terminus of chain A. Supplementary basic amino acids at B31 and B32 shift protein pI values towards physiological pH, while neutral amino acid residues at A22 prevent deamidation of the asparagine (A21) residue. In this way, we obtained chemically stabile, soluble formulations of pH 4 with prolonged action profiles. To our surprise, we found that such modifications not only extend the drugs’ duration, but also result in fast onset.

## Materials and methods

### Ethics statement

All procedures involving animals were performed in compliance with the relevant European and Polish guidelines. The studies were approved by II Local Ethics Committee at the Medical University of Warsaw (Żwirki i Wigury 61 Str., 02–091 Warsaw). The Ethics Committee approval numbers are as follow: 13th dated on 18.03.2008 (GKR), 18/2009 dated on 23.06.2009 (GEKR) and 39/2009 dated on 15.12.2009 (AKR, SR). All study procedures were designed to conform to accepted practices and to minimize or avoid causing pain, distress, or discomfort of the animals. The animals were maintained under a standard laboratory condition. The animals were kept in well ventilated rooms, controlled in respect to air temperature, relative air humidity and artificial lighting to provide an alternating 12-hour light/dark cycle. The rats were maintained on standard laboratory rodent diet and water. All animal studies were carried out according to international guidelines. After acclimatization and inducing diabetes the pivotal studies were performed. Animals were regularly observed in respect to behavior, clinical signs. During the studies none of the animals became ill or died prior to the experimental endpoint. Good health of the animals was also confirmed by the macroscopic and histopathology examinations after humane euthanasia at the planned time-point of the studies. There was no necessity to withdrawn ill animals from the experiments. After the experiment all animals were humanely sacrificed (decapitation).

### Construction of the expression plasmid pIBAINS

**Fig 2:**
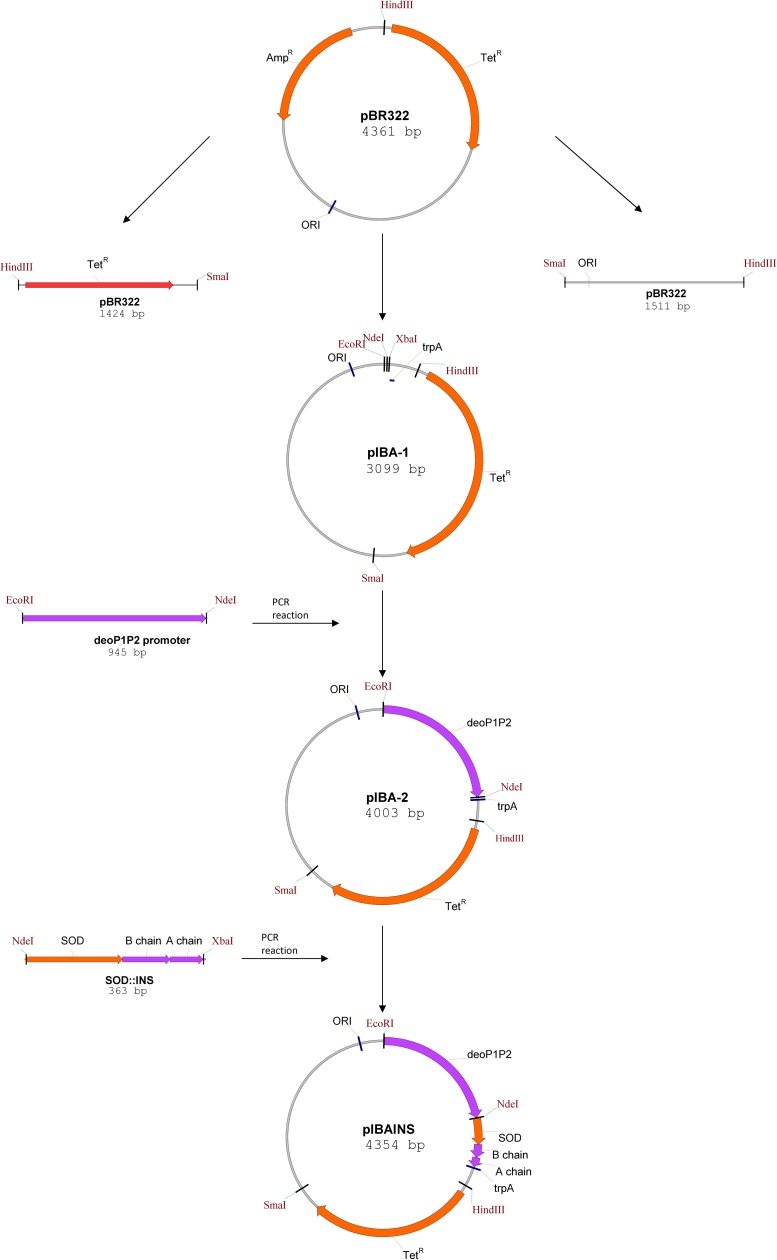
The construction scheme for the expression plasmid pIBAINS. The resulting expression plasmid pIBAINS contains a hybrid protein SOD::INS gene under the control of the *deoP1P2* promoter. Abbreviations used: Amp^R^, ampicillin resistant; Tet^R^, tetracycline resistant; trpA—transcription terminator trpA; SOD, superoxide dismutase gene; A chain, B chain—chains of human insulin gene; ORI, replication origin.

The expression plasmid pIBAINS is a derivative of plasmid pBR322 [ATCC: 31344]. Briefly, pIBA was prepared by ligation of two *Hind*III/*Sma*I fragments and amplified by PCR, using pBR322 plasmid as a template. Then, exploiting a PCR method, 63 nucleotides with short molecular cloning sites and a synthetic transcription terminator trpA (Pharmacia Biotech 1995) were inserted into this plasmid. The resulting plasmid pIBA-1 was cleaved with *EcoR*I/*Nde*I and then a 945 bp PCR fragment comprising the *deoP1P2* promoter (also digested with *EcoRI/NdeI*) was ligated in the clockwise orientation to give the pIBA-2 plasmid. The sequence of the *deoP1P2* promoter region was amplified from chromosomal DNA of *E*. *coli* K12 strain based on the nucleotide sequence from the gene database [GenBank: AP009048]. The pIBA-2 plasmid was further treated with *Nde*I/*Xba*I and ligated with 363 bp *Nde*I/*Xba*I insert, comprising a hybrid protein SOD::INS [32]. The codon usage of the hybrid protein gene was optimized for expression in *E*. *coli*. The resulting plasmid pIBAINS carries resistance to tetracycline. Transcription initiation is under control of the *E*. *coli deoP1P2* promoter. The constructed plasmid pIBAINS was used to transform the *E*. *coli* 20 strain obtained in our Institute. Fig 2 illustrates the strategy employed for the construction of the expression vector pIBAINS.

### Construction of genes of insulin analogs

Construction of new insulin analogs that comprise a sequence encoding an insulin of the following formula: L-B-X-A is described in our patent [33]. In the formula, L is a leader peptide represented by the modified SOD gene, B is human insulin chain B or a related analog, X is a short peptide, i.e. Lys-Arg or Arg-Arg connecting chain B with A. A denotes the A chain of human insulin or a related analog. The human insulin gene consists of chain B, Lys-Arg short link and chain A. Lys(31B)Arg(32B) human insulin is further named as insulin KR. Insulin analogs were prepared by mutagenesis of the insulin KR gene in the pIBAINS vector. The point mutagenesis reaction was carried out using QuickChange Site-Directed Mutagenesis Kit (Stratagene). Oligonucleotide primers were specifically designed for each analog (Table 1). Four insulin analogs were constructed (Table 2). The GKR insulin gene differs from the model human proinsulin gene in that it has an additional GGT codon attached at the C terminus of chain A. As a result, the amino acid sequence of chain A is elongated at position 22 with a glycine residue. The GEKR insulin gene has an additional GGT codon for glycine attached at the C terminus of chain A at position 22 and AAC codon is replaced with GAA codon at position 3 of chain B. The AKR insulin gene has an additional GCT codon attached at the C terminus of chain A and, as a result, is elongated at position 22 with an alanine residue. The SR insulin gene differs from the model human proinsulin gene in that it has an additional TCT codon for serine, attached at the C terminus of chain A and also an AAG codon at position 31 of chain B was replaced with a CGT codon. Introduction of positively charged amino acids changes some chemical and physical properties of insulin. The most important variation is a shift of the isoelectric point in respect to unmodified human insulin from about 5.4 towards more neutral, which finally makes insulin analogs less soluble at the neutral pH.

**Table 1:** Nucleotide sequences of primers used for the point mutagenesis reaction.

Primer name	DNA sequence (altered or added codon underlined)	Name of the insulin analog
INGly1	5’ CTGGAGAACTACTGCAAT**GGT**TAAGGATCCTCTAG 3’	GKR, GEKR
INGly2	5’ CTAGAGGATCCTTA**ACC**ATTGCAGTAGTTCTCCAG 3’	GKR, GEKR
GLUG	5’ GTC**GAA**CAGCACCTGTGTGGTTC 3’	GEKR
GLUD	5’ GCTG**TTC**GACAAAACGAGGACCTGC 3’	GEKR
ALAG	5’ CAAT**GCT**TAAGGATCCTCTAG 3’	AKR
ALAD	5’ CTTA**AGC**ATTGCAGTAGTTCTCCAG 3’	AKR
SERG	5’ CAAT**TCT**TAAGGATCCTCTAG 3’	SR
SERD	5’ CTTA**AGA**ATTGCAGTAGTTCTCCAG 3’	SR
ARGG	5’ CTAAAACA**CGT**CGCGGCATCGTTGAACAG 3’	SR
ARGD	5’ CGATGCCGCG**ACG**TGTTTTAGGAGTGTAG 3’	SR

**Table 2:** Changes in amino acid sequence in new insulin analogs in reference to human insulin.

Name of the insulin analog	Additional and modified codons in new insulin analogs (position in the chain is given in brackets)
GKR	Gly(22A), Lys(31B), Arg(32B)
GEKR	Gly(22A), Glu(3B), Lys(31B), Arg(32B)
AKR	Ala(22A), Lys(31B), Arg(32B)
SR	Ser(22A), Arg(31B), Arg(32B)

### Bacterial strain

*E*. *coli* 20 is a laboratory derivative of *E*. *coli* CSH50R [ATCC: 39111] [34–36]. *E*. *coli* 20 *F*^*-*^ *λ* ^*-*^ *ara Δ(pro lac) rpsL thi recA cytR*, harboring a pIBAINS plasmid carrying the hybrid genes, was used as a host strain for expression. The expression of the SOD::INS gene in the pIBAINS vector is under the control of the *deoP1P2* promoter, which is negatively controlled by a chromosomal repressor protein, the product of the cytR gene (anti-activator for the CytR-CRP nucleoside utilization regulon) [37,38]. To effectively produce proteins under the control of the *deoP1P2* promoter, it was necessary to modify the gene sequence in the CSH50R strain. The sequence of the cytR gene was modified by site-directed mutagenesis–deletion of 145 bp from the sequence, resulting in a frame shift and creating a non-functional repressor protein. Around the places of interest within the target gene cytR, the rpsL-neo cassette was inserted via 50 bp homology arms using recombination techniques. Positive clones had the Kan^R^Str^S^ phenotype. In the next step, the rpsL-neo cassette was replaced against the modified single-stranded (ss) DNA-fragment of the target gene (sequence cytR gene carrying the deletion of 145 bp). Positive clones became Str^R^ again. This bacterial strain is Gram-negative, streptomycin resistant and was named *E*. *coli* 20 and requires proline and thiamine (2 μg/mL) for growth in a minimal medium. Concentration of proline was tested in the range 50–200 μg/mL in flasks and 600 μg/mL during fermentation in 4 L medium in a fermenter.

### Cell culture and recovery of inclusion bodies

*E*. *coli* 20 cells, harboring plasmids pIBAINS with four different insulin analog genes, were grown for 18 h at 30°C in shaking flasks containing 50 mL GMS medium [39] supplemented with tetracycline (100 μg/mL), proline (600 μg/mL) and thiamine (2 μg/mL) until the optical density (OD_600_) reached at 600 nm was about 0.5–1.0. The flasks were used as an inoculum for a 7.5 L fermenter (starting GMS medium volume 4 L). During the growth stage of the culture, 40% glucose and 12 mg/mL proline were exponentially added as the carbon source (to maintain glucose concentration range 70–120 mg/dL). The pH was controlled during the entire run by addition of 16% NH_4_OH. When OD_600_ reached about 25–35, the glucose feeding was limited until its concentration in the medium was reduced to 0 g/dL. Then, the glucose feeding was connected to the pump of acid (pH-stat control using cascade). Glucose was automatically added by the controller whenever the pH exceeded the set point (dead band was 0.01). The glucose level was maintained by the pH-stat in the concentration range of 30–50 mg/dL. After induction, the culture was grown for 6–8 h until OD_600_ was about 50–60, reaching the stationary phase of growth. Cells were harvested by centrifugation at 6,700 xg for 15 min at 4°C. The pelleted cells were suspended in 50 mM Tris-HCl (pH 7.5), 500 mM NaCl, 1mM EDTA, 0.02% lysozyme, lysed by sonication and centrifuged at 22,100 xg for 20 min. The resulting pellets were washed twice with 50 mM Tris-HCl (pH 7.5), 500 mM NaCl, and 1% Triton X-100, followed by centrifugation at 22,100 xg for 20 min to remove bacterial proteins. Finally, the pellet was washed with 50 mM Tris-HCl (pH 7.5), 500 mM NaCl buffer.

### Downstream processing of fusion proteins

The inclusion bodies containing hybrid insulin analogs were suspended individually in 12 mM NaHCO_3_ and 0.2 mM EDTA pH 12.0 buffer, and gently stirred for 30 min at room temperature. Then, fusion proteins were allowed to fold during the subsequent 18 h period with vigorous stirring and aeration. The pH was lowered to 11.2 with 2 M HCl solution. At the end of the folding period, the pH was adjusted to 9.0 with 2 M HCl. The insulin precursor was treated with citraconic anhydride in order to control enzymatic digestion required for forming insulin and prevent insulin degradation. The citraconilated folding mixture was digested with trypsin [40], according to the protein concentration previously determined by the Bradford method [41]. Further stages included chromatographic purification, successively: DEAE, Q, and then carboxypeptidase B (CPB) digestion. The sample was then purified by RP-HPLC using a Kromasil C8 column and eluted at a flow rate of 4 mL/min with a gradient of 0–22% buffer B (50% isopropanol in H_2_O, 1.5 mS ammonium sulfate, pH 3.0) for 40 min with UV detection at 254 nm. The fractions were collected during a RP-HPLC run. The proteins after RP-HPLC were precipitated with zinc acetate and lyophilized.

### Pharmaceutical formulations

All insulin analogs were formulated as aqueous, clear and colorless solutions that contained 100 U/mL of the insulin analog (1U = 6 nmol; 1 unit contains the same number of moles of analog as 1 international unit of human insulin), 16 mg/mL of glycerol, 2.7 mg/mL of m-cresol and 30 mg/mL of zinc. The pH 4.0 was adjusted by addition of aqueous solutions of HCl and NaOH. Pharmaceutical formulations were stored at 5°C and 37°C and evaluated by chromatographic methods at appropriate intervals to assess chemical stability. Derivatives of insulin analogs, which are products of hydrolysis and other transformation reactions during storage, were monitored by RP-HPLC. Covalent dimers and aggregates of insulin analogs, which are formed due to intermolecular chemical reactions during storage, were evaluated by a Pharmacopeia SEC (size-exclusion chromatography) assay [42].

### Physicochemical characterization

#### Molecular weight

Molecular weight measurements were performed in reflector positive ion mode with the use of a matrix-assisted laser desorption/ionization mass spectrometer (4800 Plus MALDI-TOF/TOF MS, AB SCIEX, Framingham, USA). Alpha-cyano-4-hydroxycinnamic acid (CHCA) from Sigma-Aldrich (Munich, Germany) was used as a MALDI matrix. External calibration was achieved with a 4700 proteomic analyzer calibration mixture provided by AB SCIEX. Data Explorer Software, Version 4.9 was used to process the acquired spectra.

#### Amino acid sequencing

An amino acid sequence of all the analogs was established, based on the UHPLC-ESI-Q-TOF measurement system (Dionex UltiMate 3000 Rapid Separation LC (RSLC), Thermo Fisher Scientific Inc, combined with micrOTOF-Q II™ ESI-Qq-TOF MS, Bruker, USA). Prior to analysis, the insulin analogs were reduced with tributylphosphine and alkylated with iodoacetamide. The mobile phase consisted of 1% acetic acid: acetonitrile. The analytes were eluted gradiently at a flow rate of 0.15 mL/min. Phenomenex Jupiter 4u Proteo 90A, 1 x 150 mm, 4 μm column and Security Guard precolumn were used. The column temperature was set to 45°C. MS calibration was performed with ESI-L Low Concentration Tuning Mix (Agilent, USA). Analyses of the MS/MS spectra of chains A and B were performed with BioTools (Version 3.2, BrukerDaltonics).

#### Isoelectric point (pI)

The isoelectric point (pI) of insulin analogs was determined on a PA 800 plus Analysis System from Beckman Coulter, according to the manufacturer’s procedure [43], with the exception that the final concentration of urea in the isoelectric focusing (IEF) gel was increased to 4 M. The cIEF reagents, neutrally coated capillary, gel, and pI peptide markers were obtained from Beckman Coulter. Isoelectric point (pI) calculations were performed using a specially written program based on the model proposed by Sillero [44], using amino acid dissociation constants and the number of disulphide bridges within the protein molecule.

#### RP-HPLC

RP-HPLC analysis was carried out on an Alliance instrument (Waters Corp.), employing an ACE C18-300 column (5 μm, 4.6 x 250 mm, Advanced Chromatography Technologies). Elution was performed with a mobile phase A: 0.2 M Na_2_SO_4_ buffer solution (adjusted to pH 2.3 with H_3_PO_4_), acetonitrile, 82:18 (v/v), B: 0.2 M Na_2_SO_4_ buffer solution (adjusted to pH 2.3 with H_3_PO_4_), acetonitrile, 50:50 (v/v); (0–60 min) isocratic elution at A/B = 78/22; (60–83 min) linear change to A/B = 51/49; (83–84 min) linear change to A/B = 78/22; flow rate 1.0 mL/min; absorbance monitoring at 214 nm.

#### SEC

SEC analysis was carried out on an Alliance instrument (Waters Corp.) employing an Insulin HMWP column (7.8 x 300 mm, Waters Corp.) set to room temperature. Elution was performed with a mobile phase consisting of 15% (v/v) acetic acid, 20% (v/v) acetonitrile, 0.65 g/L L-arginine (240 mM) with a flow rate 0.50 mL/min for 35 min.

### Biological activity

The biological activity of the new IBA’s insulin analogs was evaluated by *in vivo* studies on rats. Experiments for each of the analogs were conducted independently and their main purpose was to confirm the hypoglycemic activity of the new analogs in comparison to the reference products–a commercially available long-acting insulin analog, Lantus (insulin glargine) or Levemir (insulin detemir). Bioactivity evaluation was performed on young adult (10–12 weeks of age), Wistar Albino Glaxo (WAG) rats of both sexes c.a. 300 g of body weight (b.w.) each, which were randomly divided into study groups that received single or multiple doses of the tested analogs. Experimental hyperglycemia was induced by streptozotocin injections according to the Nakhoda-Wong method [45]. 16 h prior to administration of streptozotocin, the animals were deprived of access to feed and allowed free access to water. Animals with induced diabetes, proved by three day glucose determination, were qualified for the pivotal experiments and randomly divided into study groups (test and control–negative and positive). Evaluation of the hypoglycemic effect was performed in comparison to a positive control–commercially available long-acting insulin analogs (insulin glargine or insulin detemir). Negative control groups both in single and multiple studies consisted of animals treated with physiological salt solution (0.9% NaCl), 10 μl/100 g b.w. They were independently maintained for each analog’s experiment.

During each study the amounts of chow, water intake and body weight were regularly controlled. An example of such results obtained for the SR sub-study after the multiple doses is supplied in the Supplementary materials (Tables A-C in S1 File).

#### Single dose studies

**Fig 3:**
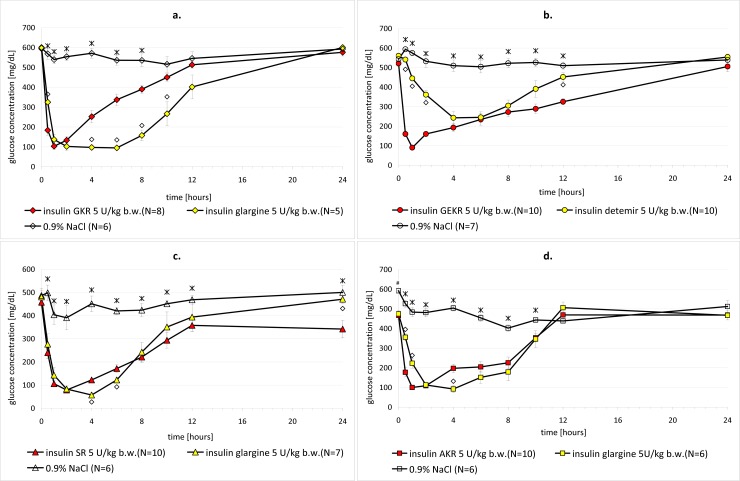
**a-d. Glycemia profiles after single dose administration of tested insulin analogs (GKR, GEKR, SR and AKR), positive (insulin glargine or insulin detemir) and negative (0.9% NaCl) control in streptozotocin-induced diabetic rats. Mean glycemia (± SEM).** Star–statistical significance (p<0.05) confirmed in a Newman-Keuls test between one of the tested groups (GKR, GEKR, SR or AKR) and a negative control group (0.9% NaCl) at the individual sampling time point. Diamond–statistical significance (p<0.05) confirmed in a Newman-Keuls test between one of the tested groups (GKR, GEKR, SR or AKR) and a positive control group (insulin glargine or detemir) at the individual sampling time point. N–Number of tested animals per group.

In the single dose studies, animals with severe diabetes (induced by streptozotocin in doses of 38–40 mg/kg b.w.) were used. The mean values of glycemia in each group always exceeded 400 mg/dL. The sample size (N) for the tested groups was 8 or 10 rats (details in Fig 3). Glycemic profiles, after single administration of the tested analogs, were compared with profiles of the physiological salt solution as well as the reference insulins. In the case of GKR, SR and AKR, insulin glargine was chosen as a comparator, while in the GEKR experiment, insulin detemir was used. Both test and reference analogs were administered to diabetic rats subcutaneously at single doses of 5 U/kg b.w. The products were administered directly from vials with a Hamilton syringe with the calculation of the applied doses in units (U) per kg of rat b.w. Blood for glucose determination was collected at predefined time points from the tail vein of individual animals. Glucose concentration was measured using the glucometer Accu-Check Active (Roche Diagnostics Corp.) with a measuring range of 10–600 mg/dL. The glucose concentration measurement was performed just before administration and then 30 min 1, 2, 4, 6, 8, 10, 12, and 24 h after administration.

#### Multiple dose studies

**Fig 4:**
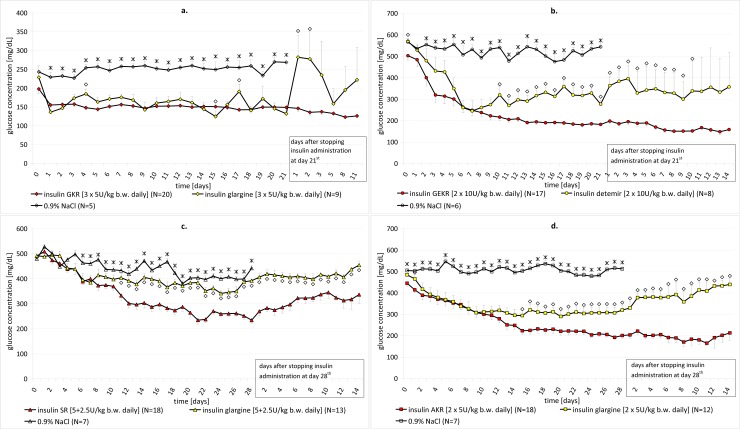
**a-d. The influence of multiple dose administration of tested insulin analogs (GKR, GEKR, SR and AKR) positive (insulin glargine or insulin detemir) and negative (0.9% NaCl) controls on glucose concentration in streptozotocin-induced diabetic rats, together with the period after termination of analogs administration. Mean glycemia (± SEM).** Star–statistical significance (p<0.05) confirmed in a Newman-Keuls between one of the tested groups (GKR, GEKR, SR or AKR) and a negative control group (0.9% NaCl) at the individual sampling time point. Diamond–statistical significance (p<0.05), confirmed in a Newman-Keuls test during the administration period or in a t-test after stopping insulin administration, between one of the tested groups (GKR, GEKR, SR or AKR) and a positive control (insulin glargine or detemir) at the individual sampling time point. N–Number of tested animals per group.

In the multiple dose studies, analogs were administered to rats with severe (in GEKR, SR and AKR experiments) or mild diabetes (GKR experiment). To induce severe diabetes STZ doses of 38 or 40 mg/kg b.w. were used. The mild kind of diabetes with a minimum glucose level of 140 mg/dL was induced by streptozotocin in doses of 32 mg/kg b.w. The sample size (N) for the tested groups ranged from 17–20 animals (details in Fig 4). The tested analogs were administered for 21 (GKR, GEKR) or 28 (SR, AKR) days in doses and frequency assuring the maintenance of glucose levels close to normal values. The same doses and frequency was established for reference groups in the same part of the study. As in single dose studies, insulin glargine was chosen as a reference product, only in the GEKR experiment was it insulin detemir. The daily dose of analogs ranged from 7.5 to 20 U/kg b.w. and was divided into 2 or 3 doses. Insulin GKR was administered three times a day at dosage of 5 U/kg b.w. The daily dose of GEKR, and AKR was divided into two equal doses: 10 U/kg b.w. or 5 U/kg b.w., respectively. SR was dosed at 2.5 and 5 U/kg b.w. daily. As in the single dose studies, physiological salt solution was a negative control group.

As described previously for single dose studies the products were administered directly from the vials using the Hamilton syringe, with the calculation of the applied doses in U/kg of rat b.w. every day that the tested products were administered. The same procedure of blood collection and glucose determination was used as in single dose studies. Glycemia in the steady-state was controlled once-a-day in the morning before the first daily dose of an analog during the whole drug administration period. Observation was continued and a glycemia measurement was performed for up to 11 days (in case of GKR) or up to 14 days (in case of GEKR, SR, AKR) after stopping the analog administration.

#### Statistic procedures

Statistical analysis for the glycemic effect was performed independently for each of the experiment. The normality distribution was assessed using the Shapiro-Wilk test and univariate variance analyses were performed. In the single-dose regimen and in the multiple-dose regimen, during the treatment, the glucose level was compared within the tested groups (novel insulin analog, reference insulin and negative control) at each sampling time using ANOVA at α = 0.05. When the effect was statistically significant, further evaluation was performed by the Newman-Keuls test to determine the individual effects. The glycemic results, obtained after discontinuation of drug administration, were processed statistically between the test and reference groups, using a t-test for independent samples. In all cases, p≤0.05 was taken as the minimum level of statistical significance. The processed results are presented in Figs 3 and 4 indicating the groups between which certain statistically significant differences were stated. Microsoft Office Excel 2010 and Statistica 10 were used to conduct the descriptive and comparative statistical calculations.

#### Limitations

The aim of the paper was to present the whole process of developing new insulin analogs from construction of a new plasmid to pharmaceutical formulation. Researches were conducted over many years and the testing was refined over time. *In vivo* animal experiments were performed independently for each analog. Their main purpose was to confirm the hypoglycemic activity of the new analog in comparison to the reference product. Hence, the inconsistent doses of analogs and other differences in the *in vivo* studies methodology are presented in the paper. Taking these aspects into account, the bioactivity studies should be seen as a proof of concept.

## Results and discussion

**Fig 5:**
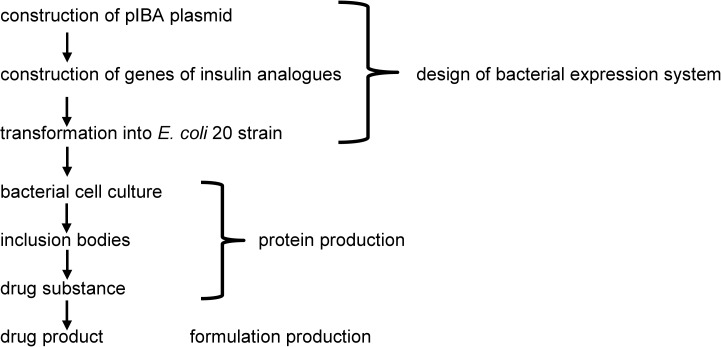
The scheme for development and production of new analogs.

Insulin KR (Lys(B31)Arg(B32) human insulin) was the first long-acting analog produced and tested in our Institute. The structure of this analog was similar to human insulin (Fig 1) with the exception that two amino acids, Lys(B31) and Arg(B32), were added at the end of chain B. Physicochemical properties, biological activity and stability were investigated at an early stage of protein development [39,46]. Although the analog exhibited biological activity, it failed to show chemical stability. During stability testing, fast deamidation at Asn(A21) was observed in pharmaceutical formulations. Insulin KR and deamidated insulin KR are two different chemical entities with potentially different biological activity, immunogenicity and toxicity. Therefore research on further development of insulin KR was terminated and new analogs with small additional amino acid at the end of chain A were designed and produced. Herein, we present the whole process of developing new insulin analogs from construction of a new plasmid to pharmaceutical formulation. For better clarity, this process can be divided into three main steps: design of the bacterial expression system, production of the protein and the pharmaceutical formulation (Fig 5).

Biosynthesis of four new insulin analogs, coded GKR, GEKR, AKR and SR, required construction of a new expression plasmid and exploitation of a new *E*. *coli* 20 strain as a host. Although the insulin chain B elongation effect has been well documented in literature, introduction of additional amino acids to the chain A N-terminus, preserving drug stability, was recently patented by our Institute [33]. All applied structural modifications were also confirmed by NMR method [47], not all data were published. In the final step, the chemical stability and biological activity of the new analogs were confirmed. The described analogs combine both a rapid onset and a prolonged time of action. Such features of insulin derivatives are unique and have never been published before.

### Design of the bacterial expression system

The construction of the insulin expression plasmid (pIBAINS) based on pBR322 plasmid is illustrated in Fig 2. The DNA sequence encoding the insulin precursor contains a modified gene fragment of human superoxide dismutase Cu/Zn (SOD) responsible for the efficiency and reconstruction of higher order structures. The N-terminal SOD leader sequence permits folding and the correct disulfide bond formation of the hybrid polypeptide [32]. All analog genes are under *deoP1P2* promoter control, which leads to a high expression level in *E*. *coli* [34]. We constructed four plasmids expressing different insulin analogs: GKR, GEKR, AKR, SR. Using genetic engineering methods, selected amino acids were added or modified to change the isoelectric point towards more neutral. The obtained analogs differ from human KR insulin by addition or substitution of codons as described in the experimental section. The presence of the correct inserts was verified by DNA sequencing. The introduced changes resulted in preservation of biological activity, a shift of the isoelectric point towards more neutral and finally, prolonged activity of the new insulin derivatives.

### Protein production and analysis

The newly constructed plasmids were transformed into *E*. *coli* 20 competent cells and expressed intracellularly. The *E*. *coli* 20 strain was constructed in the Institute of Biotechnology and Antibiotics, based on *E*. *coli* CSH50R [ATCC 39111] as described previously. The expressed fusion proteins were found to accumulate as insoluble inclusion bodies in the cytoplasm, as expected. The insoluble inclusion bodies, containing insulin analog fusion proteins, were processed to form insulin analogs as described in the experimental section. Molecular masses of all the described insulin analogs after downstream processing were confirmed by MALDI-TOF/TOF MS (Table 3). The molecular masses were in agreement with the calculated average molecular mass expected from the covalent structure of insulin analog molecules. Their amino acid sequence was determined based on UHPLC-ESI-Q-TOF measurements. The sequences of the two chains of insulin analogs were found to be in agreement with the sequences predicted from the cDNA (Table 3). Both theoretical (pI calc.) and experimental (pI exp.) pI values were established for the biosynthetics (Table 3). The pI values were theoretically calculated with the precision required and correlate well with experimental data.

**Table 3:** Physicochemical properties of insulin analogs.

*Protein*	*Sequence*	*MW calc*.	*MW exp*.	*pI calc*.	*pI exp*.
GKR	*chain A*: GIVEQCCTSICSLYQLENYC NG; *chain B*: FVNQHLCGSHLVEALYLVCG ERGFFYTPKTKR	6149.0	6149.0	7.3	7.1
GEKR	*chain A*: GIVEQCCTSICSLYQLENYC NG; *chain B*: FVEQHLCGSHLVEALYLVCG ERGFFYTPKTKR	6164.0	6163.9	6.4	6.7
AKR	*chain A*: GIVEQCCTSICSLYQLENYC NA*; chain B*: FVNQHLCGSHLVEALYLVCG ERGFFYTPKTKR	6163.0	6162.9	7.3	7.0
SR	*chain A*: GIVEQCCTSICSLYQLENYC NS; *chain B*: FVNQHLCGSHLVEALYLVCG ERGFFYTPKTR	6050.8	6050.8	6.4	6.5

### Pharmaceutical formulations and stability testing

Pharmaceutical formulations were prepared and characterized as described in the experimental section. The main concerns with pharmaceutical formulations of new analogs were their stability. The chemical stability of all protein pharmaceuticals has to be evaluated prior to sale to a patient. In case of insulin and its analogs, pharmaceutical formulations have to be tested in regard to formation of related proteins (various modifications of an original protein including deamidated, oxidized, cleaved, aggregated forms), covalent dimers and polymers [42], which are considered as impurities. Progressive increase in the content of the above is expected in all insulin pharmaceutical formulations [48], however, it cannot exceed safety limits. It is assumed that the formulation is stable if the monitored parameters do not exceed the limits.

The greatest deteriorating process in insulin is deamidation, where an amide side-chain of glutamine or asparagine residues is transformed to a free carboxyl group. There are at least four different mechanisms of non-enzymatic deamidation of the residues mentioned above [49,50]. The mechanism which predominates depends on many exogenous and endogenous factors including pH, temperature, buffer species, ionic strength, neighboring residues in both the polypeptide chain as well as in the 3D structure [51]. Under acidic conditions human insulin undergoes preferential deamidation at the C-terminal asparaginyl residue A21 in chain A. This reaction proceeds through initial formation of five-membered cyclic anhydride followed by hydrolysis of the anhydride to creation of an aspartyl residue [52]. The present study gives additional evidence to confirm that deamidation at A21 in insulin chain A proceeds through the cyclic anhydride, and we showed that its formation can be blocked by the addition of a small amino acid to the terminal asparagine A21. All designed analogs were formulated as aqueous, clear, solutions of pH 4.0. In such conditions, insulin KR experienced extensive chemical deterioration. The content of deamidated derivatives and other transformation products (Table 4), as well as covalent dimers and polymers (Table 5), increased dramatically by almost 60% and 20%, respectively, after storage for two months at 37°C. Therefore, new analogs with additional small amino acids (glycine, alanine or serine) at the C-terminus of chain A were designed as candidates for stable, long-acting formulations. Thus, the carboxyl group from asparaginyl residue A21, involved in a peptide bond with an additional amino acid at A22 and cyclic anhydride, could not be formed. As a consequence, deamidation at A21 was efficiently blocked and pharmaceutical formulations of pH 4.0 remained stable (Tables 4 and 5). The percentage increase in the content of related proteins and covalent dimers and polymers was observed for all tested analogs, however only insulin KR degraded at a high rate and exceeded the limits. In a summary, insulin KR was considered unstable whereas the other analogs were stable, despite a gradual increase in the content of related proteins and covalent dimers and polymers.

**Table 4:** Chemical stability of drug product of insulin analogs during storage at 37°C: Formation of related proteins (content of related proteins in total including desamido A21).

weeks	KR [%]	GKR [%]	GEKR [%]	AKR [%]	SR [%]
0	2.1	3.2	3.1	1.2	3.0
1	13.8	4.5	3.5	1.9	3.3
2	23.8	5.0	4.5	2.6	4.1
4	39.9	6.8	5.6	4.3	5.8
8	60.6	12.4	8.3	8.4	8.8

**Table 5:** Chemical stability of drug product of insulin analogs during storage at 37°C: Formation of covalent dimers and polymers (content of covalent dimers and polymers in total).

weeks	KR [%]	GKR[%]	GEKR [%]	AKR [%]	SR [%]
0	0.11	0.08	0.04	0.04	0.07
1	4.23	0.13	0.10	0.07	0.10
2	7.76	0.17	0.16	0.11	0.14
4	14.76	0.29	0.27	0.26	0.23
8	20.43	0.55	0.50	0.56	0.41

### Biological activity of insulin analogs

Biological activity of new IBA insulin analogs was evaluated by *in vivo* studies on rats. Our experiment confirmed, with statistical significance, the hypoglycemic properties of new insulin analogs coded: GKR, GEKR, SR and AKR. All tested items had prolonged action with fast onset (Fig 3A–3D). Glucose concentrations decreased 1.9-fold for SR (mean glucose value before drug administration and in the first sampling time after drug administration was 457.20 mg/dL and 239.70 mg/dL, respectively), 2.6-fold for AKR (466.20 mg/dL vs. 177.10 mg/dL), and even 3.2-fold for GKR (594.25 mg/dL vs. 183.38 mg/dL) and GEKR (521.40 mg/dL vs. 160.10 mg/dL) in the first half hour after single administration. Once applied, the three insulin analogs: GEKR (Fig 3B), GKR (Fig 3A) and AKR (Fig 3D) presented definite rapid onset of action with the maximum effect observed in the first hour. What is important, the significant hypoglycemic effect was maintained for up to 8 h in the case of GKR, 10 h for AKR and at least to 24 h after the injection of GEKR. For insulin SR, the onset of activity was relatively slower, comparing mean glucose values to other tested analogs, with its maximum effect occurring 2 h after drug administration (Fig 3C). The significant pharmacological effect of SR lasted 24 h. The glycemic profile, after the analog application, to a great extent, reflected the prolonged characteristics of insulin [53,54]. Overall, the glycemic profiles after a single dose administration of IBA analogs suggest their prolonged action similar to the reference long-acting insulin analogs (glargine or detemir). Insulin SR and AKR have the most similar profiles in comparison to insulin glargine. A few significant differences suggest faster and stronger action of AKR within the first hour, post dose (Fig 3D), and a weaker activity of SR 4–6 h after administration (Fig 3C). GEKR characterizes faster and stronger hypoglycemic action compared to insulin detemir (Fig 3B) while GKR acts weaker than insulin glargine from the fourth hour post dose (Fig 3A). Compared to commercially available bolus insulin formulations (aspart, glulisine, lispro, regular human insulin), the analogs presented herein demonstrate similar start and peak values [55,56]. What is exceptional is that their duration of action is longer than for bolus insulins. It is comparable to the time frames given for regular, long-acting and premixed insulin formulations [57].

After a few days (1 day for GKR and AKR, 2 days for GEKR and 6 days for SR) of insulin analogs multiple administration, stable glycemia values on a level significantly lower than in the groups receiving physiological salt were observed up to the end of treatment (21 days for GKR and GEKR and 28 days for SR and AKR). The effect after GKR is similar to insulin glargine within the whole dosed phase (Fig 4A). It is worth emphasizing that the glycemia level is statistically significantly lower from 10^th^ (GKR), 11^th^ (SR) and 15^th^ (AKR) day after drug administration compared to its reference group–insulin detemir (in case of GEKR) or insulin glargine (in case of SR, AKR) (Fig 4B–4D). What is particularly interesting, the statistically significant, prolonged hypoglycemic effect of all tested novel analogs was observed up to 11 (GKR) or 14 (GEKR, SR, AKR) days after discontinuation of drug administration. Fig 4A–4D demonstrate the influence of multiple dose administration of tested insulin analogs (GKR, GEKR, SR and AKR) on glucose concentration in streptozotocin-induced diabetic rats, also through the period after termination of drug administration. Even then, glycemia values remained unchanged and as stable as during the treatment period. The mean values of glucose concentrations in the subsequent days ranged from 126 to 146, 148 to 198 and 165 to 222 mg/dL in the case of GKR, GEKR and AKR, respectively, and 235 to 345 mg/dL for SR. The statistical significant effect between AKR or SR and insulin glargine within the whole period, after discontinuation of insulin administration, is proved. The same tendency is also observed for GKR and GEKR and their positive reference group, however in some sampling points the effect is not statistically evidenced, due to a smaller sample size. The differences in the profile of action between all analogs are an interesting phenomenon to be explored thoroughly. It is supposed that all designed insulin analogs precipitate in subcutaneous tissue after injection and are slowly released into the blood. Therefore the reasons for these differences must be sought in the structure and conformation of the analogs. Based on NMR structural studies and X-ray crystallography, we know that three-dimensional structures of all analogs are similar to each other and to human insulin [X-ray crystallography data not shown, NMR structural studies published partially 46,47]. The only differences found were in pathways of self-association and formation of quaternary structures (dimers, hexamers etc.) and in the lability of the C-terminus of chain B and the N-terminus of chain A.

## Conclusions

In conclusion, all the new insulin analogs were highly expressed in *E*. *coli* 20, as SOD::INS fusion proteins, in the form of insoluble inclusion bodies. Biologically fully-active analogs were purified from fusion proteins by ion exchange chromatography, refolded, digested with trypsin, citraconylated, precipitated with zinc acetate and finally purified by RP-HPLC. Elongation of chain A with a small, neutral amino acid assured the chemical stability of the insulin derivatives under acidic conditions. All the tested analogs present hypoglycemic activity after a single dose and a stable glycemic profile during prolonged treatment.

We assume that the prolonged activity of the insulin analogs results from formation of a stable deposit at the site of administration (subcutaneous tissue). Not only did the introduced modifications result in extended duration of action of the biosynthetics, but also their fast onset. Due to these unique properties, the tested insulin analogs are candidates for even less frequent subcutaneous administration than once-a-day. Moreover, they constitute a valuable alternative for the commercially available insulins and insulin analogs with prolonged action or for mixed formulations with rapid onset.

## Supporting information

S1 File Table A. Water intake changes in the subsequent days of tested insulins administration [multiple dose administration of insulin SR and insulin glargine (2 times a day; 2.5 and 5 U/kg b.w.)]. Table B. Food intake changes in the subsequent days of tested insulins administration [multiple dose administration of insulin SR and insulin glargine (2 times a day; 2.5 and 5 U/kg b.w.)]. Table C. Body weight changes in the subsequent days of tested insulins administration [multiple dose administration of insulin SR and insulin glargine (2 times a day; 2.5 and 5 U/kg b.w.)].
(DOCX)

## Abbreviations

INS: human insulin
GKR: GEKR, AKR, SR: insulin analogs
SOD: superoxide dismutase
RP-HPLC: reverse-phase high performance liquid chromatography
NPH: Neutral Protamine Hagedorn
trpA: transcription terminator
KR: Lys-Arg insulin
CPB: carboxypeptidase B
CRP: cAMP receptor protein
OD: optical density
SEC: size-exclusion chromatography
IEF: gel, isoelectric focusing gel
WAG: rats, Wistar Albino Glaxo rats
ORI: replication origin
