# Supplementary material for: Soluble insulin analogs combining rapid- and long-acting hypoglycemic properties – From an efficient E. coli expression system to a pharmaceutical formulation
Source: PLoS One. 2017 Mar 15;12(3):e0172600. doi: 10.1371/journal.pone.0172600 (PMC5351984; doi:10.1371/journal.pone.0172600)
Supplement: S1 File — Table A. Water intake changes in the subsequent days of tested insulins administration [multiple dose administration of insulin SR and insulin glargine (2 times a day; 2.5 and 5 U/kg b.w.)]. Table B. Food intake changes in the subsequent days of tested insulins administration [multiple dose administration of insulin SR and insulin glargine (2 times a day; 2.5 and 5 U/kg b.w.)]. Table C. Body weight changes in the subsequent days of tested insulins administration [multiple dose administration of insulin SR and insulin glargine (2 times a day; 2.5 and 5 U/kg b.w.)]. (DOCX) [file pone.0172600.s001.docx]

**Data from clinical observation of animals after multiple dose administration of tested insulins in comparison to reference/control groups
- representative data from study of insulin SR**

**Table A.** Water intake changes in the subsequent days of tested insulins administration [multiple dose administration of insulin SR and insulin glargine (2 times a day; 2.5 and 5 U/kg b.w.)].

| Tested product and  dose | N | Amount of water intake  (mean [mL] ± SEM) | | | | | | | | | | | | | | |
| --- | --- | --- | --- | --- | --- | --- | --- | --- | --- | --- | --- | --- | --- | --- | --- | --- |
|  |  | Fixed hyperglycemia | Subsequent days of tested product administration | | | | | | | | | | | | | |
|  |  |  | 1 | 2 | 3 | 4 | 5 | 6 | 7 | 8 | 9 | 10 | 11 | 12 | 13 | 14 |
| SR 7.5 U/kg b.w./day | 18 | 66.4 ± 1.7 | 56.1 ± 2.9 * ^ | 56.7 ± 1.6 | 61.1 ± 1.8 ^ | 56.7 ± 3.5 | 55.6 ± 2.8 | 50.8 ± 1.8 ^^ | 45.6 ± 3.3 ^ | 52.2 ± 3.5 | 45.6 ± 3.8 | 46.1 ± 4.3 | 45.0 ± 3.7 | 42.2 ± 2.3 ^^ | 38.3 ± 3.4 ^^ | 35.6 ± 4.1 ^^ |
| Glargine 7.5 U/kg b.w./day | 13 | 71.2 ± 2.6 | 48.8 ± 1.3  ## | 58.5 ± 2.5 | 63.1 ± 3.3 | 57.7 ± 4.3 | 58.8 ± 5.1 | 43.1 ± 4.7  ## | 48.5 ± 4.9  # | 54.2 ± 6.7 | 46.2 ± 3.9  # | 47.3 ± 7.8 | 46.1 ± 4.7 | 38.8 ± 3.7  ## | 36.9 ± 3.0  ## | 38.5 ± 3.2  ## |
| 0.9% NaCl 10 µl/100 g b.w./day | 7 | 64.3 ± 0.8 | 65.7 ± 0.3 | 64.3 ± 0.8 | 71.4 ± 0.7 | 67.1 ± 2.5 | 67.1 ± 1.0 | 68.6 ± 2.9 | 64.3 ± 0.3 | 58.6 ± 2.9 | 61.4 ± 5.4 | 58.6 ± 5.2 | 57.1 ± 4.5 | 63.6 ± 3.4 | 61.4 ± 3.0 | 64.3 ± 3.2 |

Significance level (Newman-Keuls test):

** p < 0.01, * p < 0.05 - SR vs. Glargine

^^ p < 0.01, ^ p < 0.05 - SR vs. 0.9% NaCl

^##^ p < 0.01, ^#^ p < 0.05 - Glargine vs. 0.9% NaCl

N - sample size

**Table A cont.** Water intake changes in the subsequent days of tested insulins administration [multiple dose administration of insulin SR and insulin glargine (2 times a day; 2.5 and 5 U/kg b.w.)].

| Tested product and  dose | N | Amount of water intake  (mean [mL] ± SEM) | | | | | | | | | | | | | | |  |
| --- | --- | --- | --- | --- | --- | --- | --- | --- | --- | --- | --- | --- | --- | --- | --- | --- | --- |
|  |  | Fixed hyperglycemia | Subsequent days of tested product administration | | | | | | | | | | | | | |  |
|  |  |  | 15 | 16 | 17 | 18 | 19 | 20 | 21 | 22 | 23 | 24 | 25 | 26 | 27 | 28 | |
| SR 7.5 U/kg b.w./day | 18 | 66.4 ± 1.7 | 35.0 ± 3.9 ^^ | 34.2 ± 3.4 ^^ | 37.0 ± 2.8 ^^ | 38.1 ± 4.4 ^^ | 31.1 ± 3.0 ^^ | 35.3 ± 4.5 ^^ | 26.7 ± 3.2 ^^ | 31.1 ± 4.2 ^^ | 28.9 ± 4.3 ^^ | 33.9 ± 4.1 ^^ | 32.8 ± 4.7 ^^ | 34.4 ± 3.3 ^^ | 37.2 ± 3.8 ^^ | 41.4 ± 3.5 ^^ | |
| Glargine 7.5 U/kg b.w./day | 13 | 71.2 ± 2.6 | 36.5 ± 4.2  ## | 36.6 ± 2.9  ## | 40.8 ± 3.1  ## | 41.9 ± 4.2  # | 35.4 ± 2.8  ## | 32.3 ± 3.6  ## | 35.4 ± 4.2  ## | 35.8 ± 3.4  ## | 35.8 ± 2.7  ## | 40.4 ± 3.1  ## | 38.5 ± 4.7  ## | 34.6 ± 3.1  ## | 40.0 ± 3.0  ## | 42.7 ± 4.6  ## | |
| 0.9% NaCl 10 µl/100 g b.w./day | 7 | 64.3 ± 0.8 | 67.1  ± 2.2 | 67.9 ± 5.5 | 60.7 ± 5.1 | 62.9 ± 6.1 | 67.1 ± 2.2 | 67.2 ± 0.2 | 64.3 ± 0.3 | 67.1 ± 2.5 | 67.1 ± 2.5 | 70.0 ± 2.4 | 70.0 ± 1.2 | 65.7 ± 0.3 | 62.9 ± 1.0 | 68.6 ± 1.9 | |

Significance level (Newman-Keuls test):

** p < 0.01, * p < 0.05 - SR vs. Glargine – no statistical significance observed in days 15-28

^^ p < 0.01, ^ p < 0.05 - SR vs. 0.9% NaCl

^##^ p < 0.01, ^#^ p < 0.05 - Glargine vs. 0.9% NaCl

N - sample size

**Table B.** Food intake changes in the subsequent days of tested insulins administration [multiple dose administration of insulin SR and insulin glargine (2 times a day; 2.5 and 5 U/kg b.w.)].

| Tested product and  dose | N | Amount of food intake  (mean [g] ± SEM) | | | | | | | | | | | | | | |
| --- | --- | --- | --- | --- | --- | --- | --- | --- | --- | --- | --- | --- | --- | --- | --- | --- |
|  |  | Fixed hyperglycemia | Subsequent days of tested product administration | | | | | | | | | | | | | |
|  |  |  | 1 | 2 | 3 | 4 | 5 | 6 | 7 | 8 | 9 | 10 | 11 | 12 | 13 | 14 |
| SR 7.5 U/kg b.w./day | 18 | 23.8 ± 0.6 | 24.5 ± 0.3 ^^ | 23.4 ± 0.8 ^^ | 23.9 ± 0.5 | 21.1 ± 0.7 ** ^ | 20.3 ± 0.5 ** | 20.3 ± 0.2 | 20.4 ± 0.5 * | 21.3 ± 0.5 | 21.3 ± 0.5 | 19.1 ± 0.6 ^ | 19.7 ± 0.6 ^^ | 18.3 ± 0.8 * ^^ | 17.4 ± 0.8 ** ^^ | 18.4 ± 1.2 |
| Glargine 7.5 U/kg b.w./day | 13 | 21.5 ± 0.8 | 23.1 ± 1.3  ## | 23.8 ± 0.6  ## | 24.8 ± 0.5  # | 24.5 ± 0.7 | 23.7 ± 0.9  # | 21.7 ± 0.8 | 23.2 ± 1.0 | 21.1 ± 0.7 | 21.5 ± 0.8 | 21.1 ± 1.7 | 20.6 ± 0.6  # | 21.0 ± 0.7 | 21.3 ± 1.1 | 21.1 ± 0.9 |
| 0.9% NaCl 10 µl/100 g b.w./day | 7 | 17.3 ± 1.0 | 19.1  ± 0.3 | 19.3 ± 0.4 | 22.3 ± 0.1 | 23.4 ± 0.3 | 20.0 ± 0.5 | 21.2 ± 0.6 | 22.0 ± 0.5 | 21.9 ± 0.5 | 20.3 ± 0.4 | 23.5 ± 0.0 | 23.0 ± 0.6 | 22.9 ± 0.4 | 23.6 ± 0.7 | 21.3 ± 0.7 |

Significance level (Newman-Keuls test):

** p < 0.01, * p < 0.05 - SR vs. Glargine

^^ p < 0.01, ^ p < 0.05 - SR vs. 0.9% NaCl

^##^ p < 0.01, ^#^ p < 0.05 - Glargine vs. 0.9% NaCl

N - sample size

**Table B cont.** Food intake changes in the subsequent days of tested insulins administration [multiple dose administration of insulin SR and insulin glargine (2 times a day; 2.5 and 5 U/kg b.w.)].

| Tested product and  dose | N | Amount of food intake  (mean [g] ± SEM) | | | | | | | | | | | | | | |
| --- | --- | --- | --- | --- | --- | --- | --- | --- | --- | --- | --- | --- | --- | --- | --- | --- |
|  |  | Fixed hyperglycemia | Subsequent days of tested product administration | | | | | | | | | | | | | |
|  |  |  | 15 | 16 | 17 | 18 | 19 | 20 | 21 | 22 | 23 | 24 | 25 | 26 | 27 | 28 |
| SR 7.5 U/kg b.w./day | 18 | 23.8 ± 0.6 | 19.7 ± 0.8 | 18.3 ± 0.7 | 16.7 ± 0.3 * ^^ | 16.9 ± 0.9 ^ | 16.5 ± 0.4 ^^ | 16.3 ± 1.0 ^^ | 17.0 ± 0.9 ^^ | 16.0 ± 0.9 ^^ | 17.7 ± 0.7 ^^ | 15.8 ± 0.7 ^^ | 16.7 ± 0.9 ^ | 16.0 ± 0.6 ^^ | 15.1 ± 1.0 ^^ | 15.7 ± 0.9 ** ^^ |
| Glargine 7.5 U/kg b.w./day | 13 | 21.5 ± 0.8 | 18.7 ± 0.3 | 19.1 ± 0.6 | 18.0 ± 0.6  # | 16.6 ± 0.7  # | 16.7 ± 0.6  ## | 17.1 ± 0.3  ## | 17.1 ± 0.9  ## | 16.9 ± 0.7  ## | 18.3 ± 0.7  # | 16.8 ± 0.8  ## | 17.2 ± 0.3  # | 16.6 ± 0.8  ## | 17.7 ± 1.0 | 19.3 ± 1.0 |
| 0.9% NaCl 10 µl/100 g b.w./day | 7 | 17.3 ± 1.0 | 19.5  ± 0.3 | 19.7 ± 1.3 | 20.6 ± 0.6 | 21.2 ± 0.5 | 24.9 ± 0.1 | 24.8 ± 0.2 | 24.6 ± 0.7 | 21.6 ± 0.5 | 24.8 ± 0.8 | 20.3 ± 1.3 | 22.9 ± 0.0 | 20.4 ± 0.2 | 21.9 ± 0.4 | 21.9 ± 0.3 |

Significance level (Newman-Keuls test):

** p < 0.01, * p < 0.05 - SR vs. Glargine

^^ p < 0.01, ^ p < 0.05 - SR vs. 0.9% NaCl

^##^ p < 0.01, ^#^ p < 0.05 - Glargine vs. 0.9% NaCl

N - sample size

**Table C.** Body weight changes in the subsequent days of tested insulins administration [multiple dose administration of insulin SR and insulin glargine (2 times a day; 2.5 and 5 U/kg b.w.)].

| Tested product and  dose | N | Body weight  (mean [g] ± SEM) | | | | | | | | | | | | | | |
| --- | --- | --- | --- | --- | --- | --- | --- | --- | --- | --- | --- | --- | --- | --- | --- | --- |
|  |  | Fixed hyperglycemia | Subsequent days of tested product administration | | | | | | | | | | | | | |
|  |  |  | 1 | 2 | 3 | 4 | 5 | 6 | 7 | 8 | 9 | 10 | 11 | 12 | 13 | 14 |
| SR 7.5 U/kg b.w./day | 18 | 277.2 ± 3.8 | 283.4 ± 4.2 | 281.7 ± 4.2 | 278.7 ± 4.1 | 279.5 ± 4.3 | 282.7 ± 4.4 | 279.6 ± 4.1 | 281.3 ± 4.3 | 282.2 ± 4.4 | 280.9 ± 4.7 | 281.7 ± 4.6 | 282.9 ± 4.3 | 283.7 ± 4.6 | 283.7 ± 4.7 | 285.8 ± 4.7 |
| Glargine 7.5 U/kg b.w./day | 13 | 267.3 ± 6.3 | 272.8 ± 6.5 | 271.5 ± 6.1 | 269.6 ± 6.5 | 271.5 ± 6.1 | 274.2 ± 6.6 | 274.4 ± 6.6 | 275.5 ± 6.3 | 274.5 ± 6.1 | 275.2 ± 6.5 | 277.9 ± 6.7 | 277.6 ± 7.0 | 278.2 ± 7.0 | 280.0 ± 7.0 | 284.4 ± 6.7 |
| 0.9% NaCl 10 µl/100 g b.w./day | 7 | 277.4 ± 12.6 | 280.1 ± 12.8 | 279.0 ± 12.4 | 278.0 ± 12.6 | 278.1 ± 12.2 | 275.0 ± 11.8 | 277.6 ± 10.6 | 277.0 ± 11.2 | 278.3 ± 11.3 | 275.9 ± 10.9 | 267.9 ± 9.0 | 266.9 ± 8.3 | 264.3 ± 7.9 | 264.3 ± 7.9 | 262.4 ± 6.7 |

Significance level (Newman-Keuls test):

** p < 0.01, * p < 0.05 - SR vs. Glargine – no statistical significance observed in days 1-14

^^ p < 0.01, ^ p < 0.05 - SR vs. 0.9% NaCl – no statistical significance observed in days 1-14

^##^ p < 0.01, ^#^ p < 0.05 - Glargine vs. 0.9% NaCl – no statistical significance observed in days 1-14

N - sample size

**Table C cont.** Body weight changes in the subsequent days of tested insulins administration [multiple dose administration of insulin SR and insulin glargine (2 times a day; 2.5 and 5 U/kg b.w.)].

| Tested product and  dose | N | Body weight  (mean [g] ± SEM) | | | | | | | | | | | | | | |
| --- | --- | --- | --- | --- | --- | --- | --- | --- | --- | --- | --- | --- | --- | --- | --- | --- |
|  |  | Fixed hyperglycemia | Subsequent days of tested product administration | | | | | | | | | | | | | |
|  |  |  | 15 | 16 | 17 | 18 | 19 | 20 | 21 | 22 | 23 | 24 | 25 | 26 | 27 | 28 |
| SR 7.5 U/kg b.w./day | 18 | 277.2 ± 3.8 | 287.8 ± 4.9 | 285.3 ± 5.0 | 284.8 ± 4.9 ^ | 285.7 ± 5.0 ^ | 288.4 ± 5.3 ^^ | 288.6 ± 5.4 ^ | 287.3 ± 5.1 ^ | 288.1 ± 5.3 ^ | 289.2 ± 5.2 ^ | 290.2 ± 5.2 ^^ | 293.0 ± 5.6 ^ | 292.8 ± 5.6 ^^ | 293.3 ± 5.3 ^^ | 292.2 ± 5.3 ^^ |
| Glargine 7.5 U/kg b.w./day | 13 | 267.3 ± 6.3 | 284.4 ± 6.8 | 284.2 ± 6.3 | 285.4 ± 6.7 # | 283.8 ± 7.0 | 287.5 ± 6.7 # | 284.8 ± 6.8 # | 282.3 ± 6.4 | 281.8 ± 6.2 | 282.0 ± 6.9 # | 281.6 ± 7.2 # | 285.9 ± 7.3 # | 287.0 ± 6.7 # | 285.0 ± 6.7 # | 283.6 ± 6.9 # |
| 0.9% NaCl 10 µl/100 g b.w./day | 7 | 277.4 ± 12.6 | 258.6 ± 6.6 | 259.3 ± 6.1 | 257.4 ± 5.8 | 259.3 ± 6.0 | 257.4 ± 5.9 | 257.0 ± 6.2 | 257.4 ± 6.2 | 258.9 ± 6.2 | 259.1 ± 6.9 | 256.4 ± 6.7 | 260.0 ± 6.9 | 258.7 ± 6.7 | 258.6 ± 7.0 | 259.1 ± 7.1 |

Significance level (Newman-Keuls test):

** p < 0.01, * p < 0.05 - SR vs. Glargine – no statistical significance observed in days 15-28

^^ p < 0.01, ^ p < 0.05 - SR vs. 0.9% NaCl

^##^ p < 0.01, ^#^ p < 0.05 - Glargine vs. 0.9% NaCl

N - sample size
